# Identification of disease phenotypes in acetylcholine receptor-antibody myasthenia gravis using proteomics-based consensus clustering

**DOI:** 10.1016/j.ebiom.2024.105231

**Published:** 2024-07-02

**Authors:** Christopher Nelke, Christina B. Schroeter, Sumanta Barman, Frauke Stascheit, Lars Masanneck, Lukas Theissen, Niklas Huntemann, Sara Walli, Derya Cengiz, Vera Dobelmann, Anna Vogelsang, Marc Pawlitzki, Saskia Räuber, Felix F. Konen, Thomas Skripuletz, Hans-Peter Hartung, Simone König, Andreas Roos, Andreas Meisel, Sven G. Meuth, Tobias Ruck

**Affiliations:** a Department of Neurology, Medical Faculty, Heinrich Heine University Duesseldorf, Duesseldorf, Germany; b Department of Neurology, Charité - Universitätsmedizin Berlin, Berlin, Germany; c Department of Neurology, Hannover Medical School, Hannover, Germany; d Core Unit Proteomics, Interdisciplinary Center for Clinical Research, Medical Faculty, University of Münster, Münster, Germany; e Department of Neuropaediatrics, Neuromuscular Centre, Universitätsmedizin Essen, Essen, Germany

**Keywords:** Myasthenia gravis, Complement, Proteomics, Consensus clustering, Complement inhibition

## Abstract

**Background:**

The clinical heterogeneity of myasthenia gravis (MG), an autoimmune disease defined by antibodies (Ab) directed against the postsynaptic membrane, constitutes a challenge for patient stratification and treatment decision making. Novel strategies are needed to classify patients based on their biological phenotypes aiming to improve patient selection and treatment outcomes.

**Methods:**

For this purpose, we assessed the serum proteome of a cohort of 140 patients with anti-acetylcholine receptor-Ab-positive MG and utilised consensus clustering as an unsupervised tool to assign patients to biological profiles. For in-depth analysis, we used immunogenomic sequencing to study the B cell repertoire of a subgroup of patients and an *in vitro assay* using primary human muscle cells to interrogate serum-induced complement formation.

**Findings:**

This strategy identified four distinct patient phenotypes based on their proteomic patterns in their serum. Notably, one patient phenotype, here named PS3, was characterised by high disease severity and complement activation as defining features. Assessing a subgroup of patients, hyperexpanded antibody clones were present in the B cell repertoire of the PS3 group and effectively activated complement as compared to other patients. In line with their disease phenotype, PS3 patients were more likely to benefit from complement-inhibiting therapies. These findings were validated in a prospective cohort of 18 patients using a cell-based assay.

**Interpretation:**

Collectively, this study suggests proteomics-based clustering as a gateway to assign patients to a biological signature likely to benefit from complement inhibition and provides a stratification strategy for clinical practice.

**Funding:**

CN and CBS were supported by the Forschungskommission of the Medical Faculty of the 10.13039/501100003484Heinrich Heine University Düsseldorf. CN was supported by the 10.13039/501100003042Else Kröner-Fresenius-Stiftung (EKEA.38). CBS was supported by the 10.13039/501100001659Deutsche Forschungsgemeinschaft (DFG–10.13039/501100001659German Research Foundation) with a Walter Benjamin fellowship (project 539363086). The project was supported by the 10.13039/501100014690Ministry of Culture and Science of North Rhine-Westphalia (MODS, “Profilbildung 2020” [grant no. PROFILNRW-2020–107-A]).

**Research in context**

**Evidence before this study**

Myasthenia gravis (MG) is an autoimmune disease characterised by antibodies (Ab) that attack structures of the postsynaptic membrane. This results in impaired neuromuscular transmission and fatigable muscle weakness. Despite belonging to a single serological subgroup, such as anti-acetylcholine receptor (AChR)-Ab-positive patients, MG is subject to substantial heterogeneity. Therefore, a deeper pathophysiologic understanding is needed to enable patient stratification based on biological signatures. Novel therapeutic strategies have transformed the treatment of MG. The disease’s heterogeneity, the need for objective assessment tools, and the challenge of optimising the use of new treatments all emphasise the critical need for new insights into MG’s disease patterns.

**Added value of this study**

We used consensus clustering as an unsupervised tool to assign patients to disease phenotypes based on their biological profiles. We included 140 patients with anti-AChR-ab-positive MG from three centres and identified a distinct phenotype. This phenotype is defined by high disease severity, an increase of markers associated with complement activation, and a distinct antibody profile. Stratification based on this profile links disease severity to complement activation, making it a biomarker for identifying patients who may benefit from complement inhibition. The data suggests that patients with the here named PS3 phenotype are more likely to benefit from complement-inhibiting therapies. Evaluating membrane attack complex (MAC) in a cell-based assay could act as a surrogate marker for identifying these patients. This would improve the precision of treatment allocation.

**Implications of all the available evidence**

This study shows a link between disease severity and proteomic patterns of complement activation in MG. This knowledge can improve our understanding of the disease and help classify patients based on biological signatures.

## Introduction

Myasthenia gravis (MG) is a prototypical autoimmune disease defined by antibodies (Ab) directed against structures of the postsynaptic membrane resulting in impaired neuromuscular transmission and fatigable muscle weakness.^1^ The largest serological subgroup is constituted by Abs against the acetylcholine receptor (AChR) detected in 80–85% of patients.^1^

Across the spectrum of MG, patient stratification is based on several classification factors including, among others, age at onset (early and late onset), presence (or absence) of a thymoma, and serological status. However, even among a single serological subgroup such as anti-AChR-Ab-positive patients, MG is subject to substantial heterogeneity resulting in an unmet need for a deeper pathophysiologic understanding enabling patient stratification based on biological signatures. This need is aggravated by several factors: First, clinical presentation of MG is liable to considerable variation across patients, ranging from mild ocular symptoms to life-threatening myasthenic crisis requiring intensive care.^2^ Second, therapeutic decision making largely relies on clinical features. Conversely, clinical presentation fluctuates due to factors such as time of day or effects of symptomatic medication.^3^ Third, the development of novel therapeutic strategies, including complement inhibitors^4^ or neonatal Fc receptor (FcRn) antagonists,^5^^,^^6^ transformed the therapeutic landscape of MG. However, treatment responses are divergent. As such, in a recent phase 3 randomised controlled trial investigating the complement inhibitor ravulizumab, 40% of patients in the treatment group (as compared to 73% in the placebo group) did not reach a clinically meaningful improvement defined by a change of at least two points on the MG activities of daily living (MG-ADL) scale. Given the high number of treatment non-responders and potentially treatment-related severe adverse events, factors identifying patients that are likely to benefit from these therapies represent a knowledge gap.

Taken together, the heterogeneity of the disease, the requirement for objective assessment tools, and the challenge of optimising the use of novel therapeutics all highlight the critical need for novel insights into patterns of disease in MG. To address this knowledge gap, we chose mass spectrometry-based proteomics as an established biomarker discovery tool in serum samples and employed consensus clustering as an unsupervised tool to assign patients to disease phenotypes based on their biological profiles. Mass spectrometry-based proteomics has evolved as a powerful tool allowing for the identification of candidate biomarkers and exploration of disease pathophysiology, as evidenced by the Human Genome Project and the Human Protein Atlas.^7^ As opposed to other technologies, mass spectrometry-based proteomics enables the detection and concurrent quantification of proteins across the dynamic spectrum covering low-to highly-abundant proteins.^8^, ^9^, ^10^, ^11^, ^12^ Studying a cohort of anti-AChR-Ab-positive patients, this approach identified a MG phenotype defined by high disease severity, an increase of markers associated with complement activation and a distinct Ab profile. Stratification based on this profile provides a link between disease severity and complement activation, thereby serving as a biomarker identifying patients likely to benefit from complement inhibition.

## Methods

### Human participants

All patients were required to meet the national guidelines for the diagnosis of MG.^13^ At the time of serum sampling, all patients showed no evidence for apparent infections following clinical and serological investigations. We included 140 patients with anti-AChR-ab-positive MG from three centres specialised in the treatment of MG (University Hospital Düsseldorf, Charité—University Medicine Berlin and University Hospital Hannover). Patient management was in accordance with the standards of the German Myasthenia Society as previously reported.^2^^,^^14^ Patients were scored according to the Quantitative Myasthenia Gravis (QMG) and MG-ADL scores. The QMG score is an established 13-item scale to measure disease severity, while the MG-ADL score is an eight-question survey of MG symptoms.^15^^,^^16^ Patients were either treatment naïve or had been treated with one of the following medications: steroids, azathioprine, methotrexate, mycophenolate-mofetil. Patients with add-on therapies, such as rituximab, eculizumab or cyclophosphamide were excluded from the study. Patients with a known thymoma (n = 30) received thymectomy at least 6 months before study inclusion. A total of 23 patients had no thymoma but received thymectomy at least 6 months before study inclusion. The cutoff between early- (EOMG) and late-onset (LOMG) MG was set at 50 years.^17^ Participants information on sex, age, and race was self-reported. Information on socioeconomic status was not collected.

### Ethics

The study was conducted in accordance with the Declaration of Helsinki and approved by the ethics committees of the participating clinics (Heinrich-Heine University Duesseldorf EA1/281/10, Charité Berlin EA1/144/21 and University Hospital Hannover 9741_BO_S_2021). All patients signed written informed consent before serum samples were acquired.

### Biomaterial

All serum samples were cryopreserved at −80 °C prior to analysis according to the predefined standard operating procedure at the local biobanks. For mass spectrometry-based analysis, serum samples were transferred on dry ice to the Core Unit Proteomics of the University of Münster (Head: Prof. Dr. Simone König).

### Lysate generation and processing for proteomic analysis

200 μL of each serum sample were depleted using the ProteoMiner kit (Bio-Rad Laboratories Inc., Hercules, CA, USA). This subproteome was placed in Pall Nanosep® 10 K Omega filter units (10 kDa cut-off; Pall, New York, USA) and centrifuged (12,500 g, room temperature). The analyte was washed adding 100 μL urea buffer (8 M urea, 100 mM Tris Base) to the filter unit and centrifuging. For reduction (45 min), 100 μL 50 mM dithiothreitol in urea buffer were added to the filter unit. Subsequently, the unit was centrifuged again, and the sample was rinsed with 100 μL urea buffer. For alkylation, 50 mM iodoacetamide in urea buffer was placed into the filter unit. Incubation proceeded in the dark for 30 min at room temperature. Following centrifugation and rinsing twice with 300 μL 50 mM NH_4_HCO_3_ containing 10% acetonitrile (ACN) in urea buffer, 200 μL 0.01 μg/μL trypsin in 50 mM NH_4_HCO_3_ containing 10% ACN were added to the filter unit. Incubation proceeded at 37 °C overnight. Peptides were collected by rinsing the filter thrice with 5% ACN/0.1% formic acid (FA) followed by centrifugation. Samples were dried using a Speedvac (Thermo Fisher Scientific, Waltham, MA, USA) and redissolved in 10 μL 5% ACN/0.1% formic acid.

### Mass spectrometry-based proteomics

0.5 μL of peptide solutions were analysed by reversed-phase chromatography coupled to ion mobility mass spectrometry with Synapt G2 Si/M-Class nano-ultra performance liquid chromatography (UPLC) (Waters Corporation, Milford, MA, USA) using PharmaFluidics C18 μPAC columns (trapping and 50 cm analytical; PharmaFluidics, Ghent, Belgium), as previously described.^18^

Data were analysed using Progenesis for Proteomics (Waters) and the Uniprot human database. One missed cleavage was allowed, carbamidomethylation was set as the fixed and methionine oxidation as the variable modification. A shortlist of the protein output was created by demanding protein assignment by at least two peptides, a fold value of at least 2 and analysis of variance (ANOVA) P ≤ 0.05. Quality controls (profile plots) were generated with Perseus v1.6.14.0.

### Machine learning, consensus partitioning and clustering

The aim of consensus partitioning was to identify robust subgroups of patients based on biological information. For this purpose, we transformed the full proteomic dataset into a numerical matrix. Next, we performed quality control and data cleansing using the adjust_matrix function for the following steps:

1. Rows in which >25% of the samples have missing values were removed;
2. Impute missing values using the impute.knn function from the R package impute (v. 3.17);
3. In every matrix row, values larger than the 95th percentile or less than the 5th percentile were replaced by corresponding percentiles;
4. Zero variance rows were removed;
5. Rows with variance less than the 5th percentile of all row variances were removed.

As top-value method, we employed the ATC (ability to correlate to other rows) method as proposed by the authors of the *cola* package.^19^ Partitioning was performed to classify samples into *k* distinct subgroups with a given *k*. We employed spherical k-means as a modification of classical k-means. Here, cosine similarity was used as distance measurement as this distance is more efficient for separating high-dimensional datasets and provides higher robustness to technical noise. Clustering was performed by measuring the Euclidean distance. The optimal number of subgroups was determined by a set of predefined rules as proposed by the *cola* package.^19^ Application of the following set of rules suggested that k = 4 yields the optimal number of subgroups (Supplementary Fig. S1a):

1. All *k* with Jaccard index larger than 0.95 were removed because increasing *k* does not provide enough extra information.
2. For *k* with 1-PAC score larger than 0.9, the maximum *k* was taken as the best *k*. Other *k* were marked as optional best *k*.
3. If the second rule was not fulfilled, the *k* with the majority vote among the highest 1-PAC score, the highest mean silhouette, and the highest concordance was taken as the best *k*.

With this approach, 139 of 140 samples were assigned confidently to one of the PS (Supplementary Fig. S1b).

### Immunogenomics and B cell receptor (BCR) repertoire analysis

Immunogenomic analysis of the BCR repertoire was performed in cooperation with Azenta Life Sciences (Leipzig, Germany). Peripheral blood mononuclear cells (PBMCs) were shipped at −80° on dry ice for downstream processing. 10 × 10^6^ cells were analysed per sample. Total RNA was extracted from fresh frozen cell pellet samples using the Qiagen RNeasy Plus mini kit following the manufacturer’s instructions (Qiagen, Hilden, Germany). For library generation, RNA samples were quantified using the Qubit 2.0 Fluorometer (Life Technologies, Carlsbad, CA, USA) and RNA integrity was controlled using the Agilent TapeStation 4200 (Agilent Technologies, Palo Alto, CA, USA).

Bulk immunoprofiling libraries were prepared as following. Briefly, cDNA was generated from total RNA using the Takara SMARTer RACE 5’/3’ kit. Primers located in constant regions of IH/IL genes, combined with primers with SMART oilgo, were used to enrich full length IH/IL genes. Limited cycle PCR amplification was used to add Illumina platform compatible adapters. The sequencing libraries were validated on the Agilent TapeStation (Agilent Technologies, Palo Alto, CA, USA), and quantified by using a Qubit 2.0 Fluorometer (Invitrogen, Carlsbad, CA) and qPCR. The sequencing libraries were clustered and loaded on the Illumina MiSeq instrument according to manufacturer’s instructions. The sample were sequenced using a 2 × 300 paired-end (PE) configuration. Image analysis and base calling were conducted by the MiSeq Control Software (MCS) on the MiSeq instrument. Raw sequencing data (.bcl files) generated from Illumina MiSeq was converted into fastq files and de-multiplexed using Illumina’s bcl2fastq software. One mismatch was allowed for index sequence identification. After investigating the quality of the raw data, sequence reads were trimmed to remove adapter sequences. The trimmed reads were mapped against IMGT database to find the best germline V(D)J gene matches, and CDR1, CDR2, CDR3 variable region sequences. To analyse CDR3 amino acid usage frequency, CDR3 sequences were clustered according to similarity (threshold: 0.8).

Raw sequence reads were processed using built-in preset (Generic BCR amplicon) of the MiXCR software pipeline (https://mixcr.com/). In house custom python codes were used to separate MiXCR pre-processed reads by matching them to the sequences of different immunoglobulin heavy chains (IgA, IgD, IgE, IgG and IgM) and light chains (Igκ and Igλ). Each pre-processed sequences are post-analysed using in house custom python and R codes, VDJtools (https://vdjtools-doc.readthedocs.io/en/master/), and immunarch (https://immunarch.com/index.html) to evaluate Ig distribution across the BCR repertoire, Ig heavy chain variable (IGHV) gene arrangement, Ig heavy chain joining (IGHJ) gene arrangement, V-J arrangement of the IGHV and IGHJ genes, clonality, and hyperexpanded clonal composition.

### Cell-based assay

Primary human muscle cells (PHMCs) were collected and purified from patients receiving anterior cruciate ligament reconstruction after obtaining written consent at the University Hospital Düsseldorf. Patients were required to have no autoimmune disorders or infectious diseases at the time of operation. Muscle attached to the semitendinosus tendon was used for purification of PHMCs. Briefly, we used a CD56 Ab (clone N901, Beckman Coulter, RRID: AB_130791) combined with magnetic separation (Miltenyi) for PHMC purification as previously described.^20^^,^^21^ CD56 is expressed by myogenic cells as surface marker, allowing their isolation.^20^^,^^21^ Cells were cultivated on 6-well plates coated with laminin 521 (PELO Biotech). For all experiments, we used differentiated PHMCs by growing cells to full confluence. Formation of myotubes was confirmed by light microscopy. For the cell-based assay, 10 × 10^6^ cells were incubated with monoclonal Abs at a concentration of 10 μg/mL. For the treatment with serum, 10 × 10^6^ cells were incubated with NHS containing 25% v/v of the corresponding serum of either MG or HC. MAC formation was allowed to proceed for 6 h at 37 °C. Cells were fixed with 2% paraformaldehyde at room temperature for 10 min. Cells were stained with an anti-C9 neoantigen Ab (Hycult, Cat# HM2264) for 30 min at room temperature, followed by washing and secondary staining. The number of PHMCs binding the anti-C9 neoantigen Ab was quantified by gating for PHMCs as percentage of all cells using flow cytometry (CytoFLEX, Beckman Coulter). The C9 neoantigen is the C9 portion of the membrane attack complex (MAC) allowing for the identification of this structure. This antibody was validated by comparing positive and negative biological samples and by omitting the primary antibody.

### Visualisation

Figures were created using Adobe Illustrator (version 2023) and Servier Medical Art. Heatmaps were created using the R package Complex Heatmaps.^22^

### Statistics

Statistical Analysis was performed using *R* 3.5.3 and Graphpad Prism 10.2.3. Data was presented as median with IQR, mean ± SD, as absolute (n) or relative frequencies (%). We used the unpaired Student’s t test to compare two groups and the ordinary one-way ANOVA test to compare more than two groups. In cases where either the assumption of a normal distribution or homogeneity of variance were not met, we used the Mann–Whitney U test to compare two groups and the Kruskal–Wallis test to compare more than two groups. The normality assumption for quantitative data was assessed by Q–Q plots. The homogeneity of variance was tested using the F test in when comparing two groups and the Bartlett’s test when comparing more than two groups. Categorial data was compared using the Fisher’s exact test. The 95% confidence interval (CI) for the median was calculated using Graphpad Prism 10.2.3 based on the binomial distribution method, with the lower and upper bounds determined by the ranks $\frac{n}{2}-1.96\;\sqrt{\frac{n}{4}}$ and $\frac{n}{2}+1.96\;\sqrt{\frac{n}{4}}$ . These ranks correspond to the positions in the ordered data giving the interval within which the true median lies with 95% confidence. The area under the curve (AUC) was determined for receiver operating characteristic curve (ROC) analysis. The specific test used is indicated in each section. Analysis was performed in an exploratory setting without a prior calculation of statistical power. The study design was exploratory with no prior statistical power calculation to determine the sample size. Patients were not randomised and the treating physicians were not blinded. We also computed the time-weighted average steroid dose, as previously reported.^23^ The denominator for this calculation was the daily steroid dose in mg. We screened the six months prior blood sampling at study baseline for all patients treated with steroids. Given the retrospective design, the time-weighted average steroid dose was only computed for patients with information on steroid usage on at least 80% of days during the assed period of time. 36 out of 79 patients had sufficient data for this analysis.

To identify differentially regulated proteins, gene enrichment analysis (GSEA) was performed using the R package clusterProfiler (v.4.3.1) with the gene ontology biological processes (GO-BP) database.^24^

### Role of funders

The funders had no influence on the study design, data collection, data analyses, interpretation, or writing of manuscript.

## Results

### Unsupervised clustering identifies protein signatures across MG

First, our goal was to determine whether MG can be grouped based on biological signatures. For this purpose, we recruited a cohort of 140 patients diagnosed with anti-AChR-Ab positive MG from three specialised centres for MG (University Hospital Düsseldorf, Germany, Charité—University Medicine Berlin, Germany and University Hospital Hannover, Germany) from 2016 to 2023. We acquired clinical metadata as well as serum samples from each patient. The time point of blood sampling was defined as baseline. For downstream analysis, we chose proteomics due to its scalability and high-throughput capabilities. Briefly, clinical data was recorded according to the standard procedures of the German Myasthenia Register as previously described.^2^^,^^14^ Here, standardised assessment forms including clinical, demographic and longitudinal data are completed at each patient visit and stored centrally. We chose this cohort as it reflects the spectrum of anti-AChR-Ab-positive MG including patients with ocular disease as well as patients experiencing severe, generalised symptoms. The clinical and demographic data of our cohort are given in Table 1. The mean age was 61.5 years (standard deviation (SD) 24) with 76 female and 64 male patients. 30 patients had a confirmed thymoma and received thymectomy at least 12 months prior to blood sampling. 58 patients were treatment naïve in respect to immunosuppressive or immunomodulatory treatments. Out of 58 treatment naïve patients, 27 (46.5%) received acetylcholinesterase inhibitors. 82 patients received immunosuppressive therapy (IST), including azathioprine, methotrexate (MTX) and mycophenolate mofetil (MMF). 79 patients (56.4%) were treated with steroids with a median dose per day of 6 mg (interquartile range (IQR) 3–9). Out of the 82 patients treated with ISTs, 75 (91.5%) also received steroids, while 7 patients (8.5%) were only treated with an IST. 4 patients (2.8%) from the total cohort were treated with steroids without an IST with a median dose per day of 10 mg (IQR 8 to 12). Patients receiving add-on therapies, such as eculizumab, ravulizumab or efgartigimod, were excluded from this cohort at baseline as the influence on the serum proteome is potentially understudied and may introduce a confounder into the dataset, given the influence of complement inhibition on the serum proteome.^25^ Two patients received intravenous immunoglobulins one and two months before baseline, respectively. No patients received plasmapheresis three months prior study inclusion. Serum samples from ten healthy controls (HCs) were used for comparison. HCs were required to have no known disease. The HCs had a mean age of 53.5 years (SD 24). Gender was balanced with five male and five female participants.

**Table 1:** Overview of the main cohort.

Clinical characteristics	Prevalence
Anti-AChR-Ab-positive, n (%)	140 (100%)
Gender^a^, n (%)	
Female	76 (54.3%)
Male	64 (45.7%)
Age at baseline (years), mean (SD)	61.5 (24)
Age at disease onset (years), mean (SD)	51 (26)
Ethnicity^a^, n (%)	White 121 (86.4%), Black 2 (1.4%), Asian 8 (5.1%), Prefer not to disclose 9 (6.4%)
Thymoma, n (%)	
No thymoma	110 (78.6%)
Thymoma	30 (21.4%)
QMG score, median (IQR, Q1–Q3)	
Baseline^b^	4 (2–7)
MG-ADL score, median (IQR, Q1 to Q3)	
Baseline^b^	5 (2–9)
Treatment, n (%)	
Treatment naïve	58 (41.4%)
Standard IST	82 (58.5%)
Azathioprine	57 (40.1%)
Methotrexate	8 (5.7%)
Mycophenolate-mofetil	17 (12.1%)
Steroid-treated patients	79 (56.4%)
Steroid dose at sampling, median (IQR, Q1–Q3)	6 (3–9)
Time-weighted average steroid dose per day, median (IQR, Q1–Q3)	6 (2.4–6)
Reported a previous history of COVID19^c^	37 (26.4%)

Ab, antibody; AChR, acetylcholine receptor; IST, immunosuppressive therapy; IQR, interquartile range; MG-ADL, myasthenia gravis activities of daily living; SD, standard deviation; Q1, first quartile; Q3, third quartile; QMG, quantitative myasthenia gravis.

a Ethnicity and gender were self-reported by the participants.

b Baseline is defined as the time of blood sampling.

c No patients had signs of a COVID19 infection at the time of blood sampling.

For downstream analysis, all serum samples were stored centrally and processed concurrently for mass-spectrometry based proteomics. Proteins were enriched using ProteoMiner to dilute high-abundance proteins while concentrating medium- and low-abundance proteins on their specific affinity ligands.^26^ After quality control, the entire dataset consisted of 60,480 individual datapoints comprising 432 proteins per patient across 140 patients. To identify patient subgroups based on their proteomic patterns, we applied consensus clustering to the full dataset. Briefly, consensus clustering summarises a consensus classification from a list of individual classifications by repeatably performing clustering on random subsets of data, hence offering increased stability compared to standard clustering.^27^ As computational framework for consensus clustering, we employed the recently developed *cola* package.^19^ It is important to note that the consensus clustering algorithm does not have access to any clinical data or metadata and clusters patients based only on their individual protein patterns. The number of partitions (in our study: number of patient phenotypes) is determined based on the stability of the final consensus cluster (Supplementary Fig. S1).

**Fig. 1:**
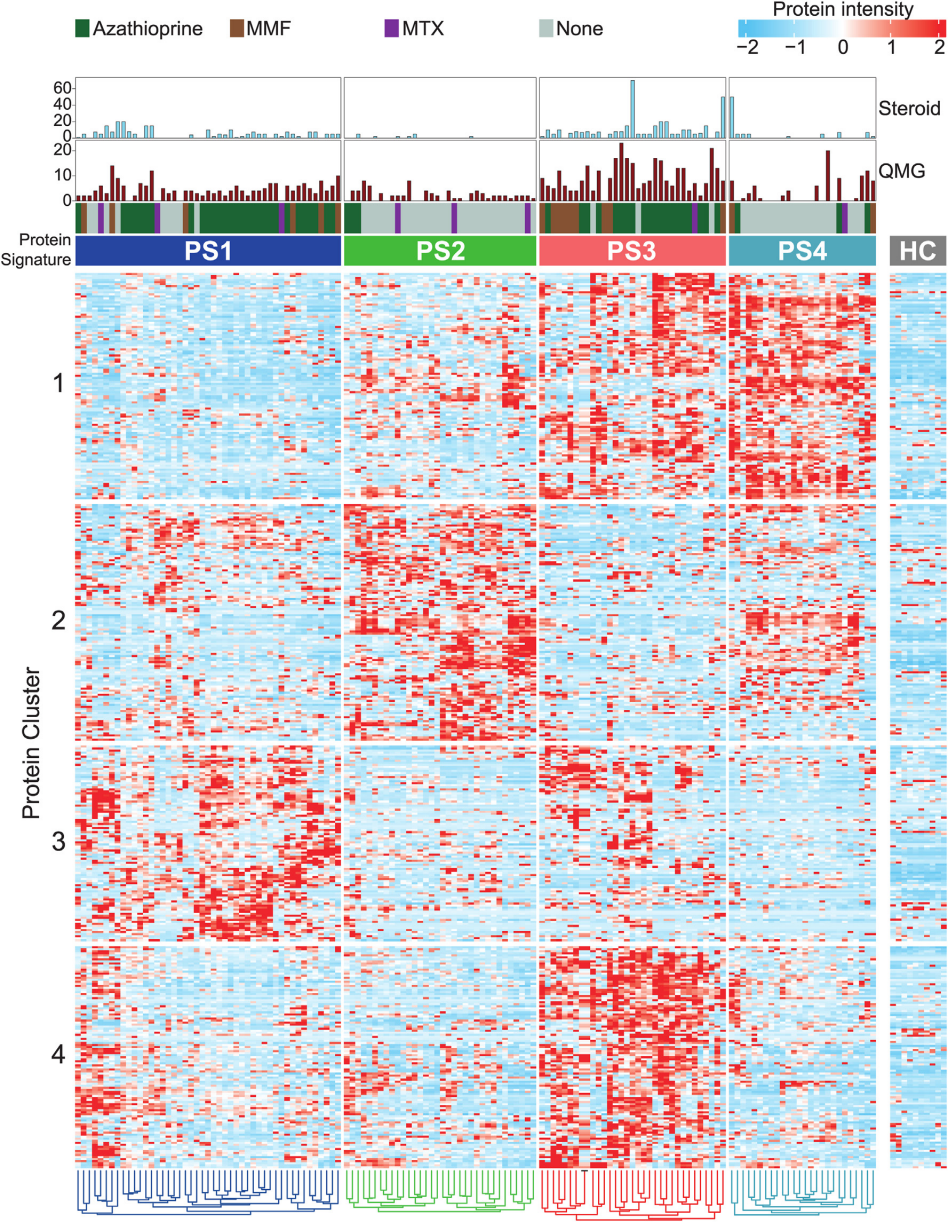
Consensus clustering of mass spectrometry-based proteomics. Heatmap displaying the serum proteome of 140 patients with anti-AChR-Ab MG and 10 HC. All detected proteins that passed quality control are shown for each patient. The normalised protein intensity is indicated by colour code. Consensus clustering was applied to group patients based on their protein signature (PS). PS1 contained 47 patients (33.5%), PS2 contained 34 (24.2%), PS3 contained 33 (23.6%) and PS4 contained 26 (18.6%). Consensus clustering determined four PS as optimal partitioning. Proteins were clustered based on correlation. These clusters were termed protein cluster. Clinical data for each patient is indicated in the top rows.

This approach determined four distinct patient phenotypes as optimal clustering based on model stability. We termed these subgroups as protein signature (PS) 1 to PS4 (Fig. 1, the full heatmap can be viewed interactively at https://masanneck.shinyapps.io/Cola-Heatmap). Each protein signature was defined by a cluster of proteins or a combination thereof. Protein clusters were defined by their spherical k-means as groups of proteins that are highly intercorrelated, e.g., protein cluster 1 is shared across PS3 and PS4 while protein cluster 2 is only present in PS2. Notably, none of the protein clusters observed in MG were found in HC. PS1 contained 47 patients (33.5%), PS2 contained 34 (24.2%), PS3 contained 33 (23.6%) and PS4 contained 26 (18.6%). Next, we superimposed clinical data onto the clustered data.

**Fig. 2:**
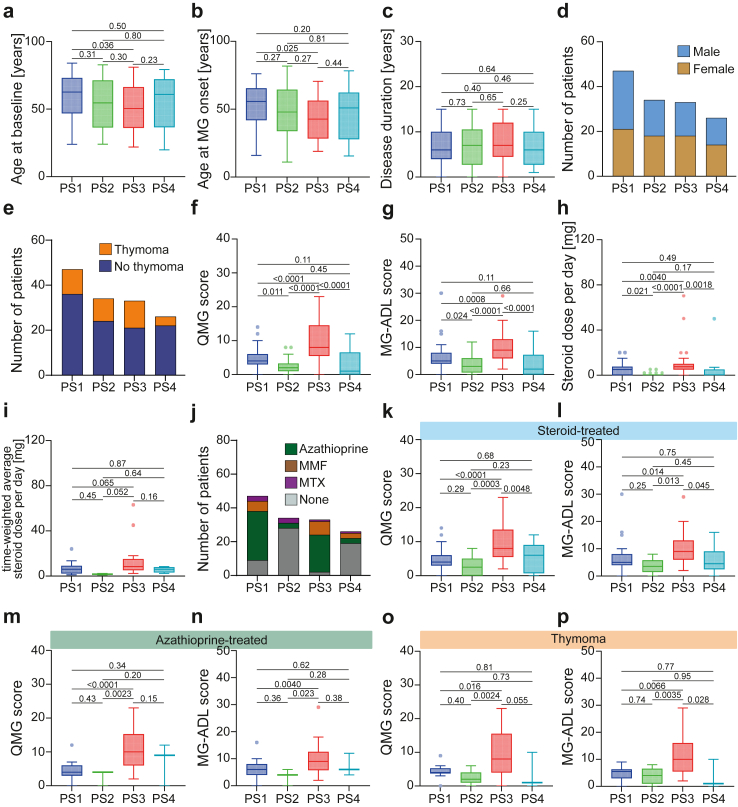
Clinical characteristics of the cohort. (a) Age in years for each group of patients stratified by their protein signature (PS) at the time of blood sampling for this study. (b) Age in years for each group of patients at the time of their individual onset of disease. (c) Disease duration in years for each group of patients defined as the time between disease onset and the time of blood sampling for this study. (d) Patient sex indicated by stacked bar plots. (e) Number of patients with or without a histologically confirmed thymoma. (f–i) Box plots indicating the QMG score, MG-ADL score, the steroid dose per day and the time-weighted average steroid dose per day. (j) The number of patients receiving the indicated immunosuppressive therapy. (k and l) Box plots indicating the QMG score and MG-ADL score for patients receiving steroids at the time of blood sampling or one month prior. (m and n) Box plots indicating the QMG score and MG-ADL score for patients receiving azathioprine at the time of blood sampling or three months prior. (o and p) Box plots indicating the QMG score and MG-ADL score for patients with a histologically confirmed thymoma. Patients received thymectomy at least 6 months before study inclusion. Groups were compared by the ordinary one-way ANOVA test, except for (d), (e) and (j). Clinical data is presented for the full cohort of n = 140 patients. Whiskers extend from the box to the minimum and maximum values within 1.5 × IQR from Q1 and Q3, respectively. These groups were compared by the Fisher’s exat test.

Intriguingly, there were no differences between individual PS groups in respect to the age at baseline (blood sampling), the age at MG onset or the disease duration (Fig. 2a–c). In line, the frequencies of EOMG and LOMG were comparable between groups (EOMG/LOMG (n, %), PS1: 20/27 (42/58%), PS2: 18/16 (53/47%), PS3: 19/14 (57/43%), PS4: 12/14 (47/53%)). There were no differences in regard to sex or the number of patients with a confirmed thymoma (Fig. 2d and e, Supplementary Fig. S2). Conversely, PS3 was defined by high disease severity as measured by the QMG score (difference between medians of the PS3 and non-PS3 groups (95% confidence interval (CI)): 5.0 (4.0–8.0), P < 0.0001, Mann–Whitney U test) and the MG-ADL (difference between medians of the PS3 and non-PS3 groups (95% CI): 5.0 (3.0–6.0), P < 0.0001, Mann–Whitney U test) score (Fig. 2f and g). At the time of blood sampling, 34 patients (72.3%) in PS1, 6 patients (17.6%) in PS2, 29 patients (87.8%) in PS3 and 10 patients (38.5%) in PS4 were treated with steroids. The steroid dose was higher for PS3 at the time of blood sampling compared to the other PS groups (difference between medians of the PS3 and non-PS3 groups (95% CI): 7.5 (4.0–7.0), P < 0.0001, Mann–Whitney U test, Fig. 2h). This effect was promoted by a number of patients in PS3 with particularly high steroid doses. Concurrently, we assessed the time-weighted average steroid dose during the six months’ period before sampling at study baseline for patients with available data (the number of available patients for analysis is indicated in Fig. 2i). The time-weighted average steroid dose was higher for the PS3 group compared to the other PS groups (difference between medians of the PS3 and non-PS3 groups (95% CI): 3.9 (0.6–7.8), P = 0.014, Mann–Whitney U test). Consistent with this, patients clustered as PS3 more frequently required ISTs compared to PS2 and PS4, while PS1 was between these patient groups (Fig. 2j). Acknowledging that the impact of different treatment strategies likely influenced the proteomic data and subgroup assignment, we matched patients based on treatments. We specifically analysed patients receiving steroids and excluded those who did not receive steroids at the time of sampling or one month prior (Fig. 2k and l). Among steroid-treated patients, those in PS3 demonstrated higher QMG and MG-ADL scores compared to other patient groups. Similarly, we repeated this analysis for patients treated with azathioprine, retaining only those who received the medication at the time of sampling or three months prior (Fig. 2m and n). Notably, patients in PS3 exhibited higher QMG and MG-ADL scores compared to counterparts in the other PS groups. Lastly, we investigated whether the presence of a thymoma influenced disease severity based on cluster assignment (Fig. 2o and p). Patients with thymoma-associated myasthenia gravis belonging to PS3 showed increased QMG and MG-ADL scores compared to those in the other PS groups. In this cohort, the QMG and MG-ADL were highly correlated (Supplementary Fig. S3).

Consequently, consensus clustering enabled the identification of MG phenotypes based on differences in their serum proteome. Notably, PS3 emerged as a phenotype characterised by a distinct clinical profile and, given the high burden of disease despite treatment, these patients might represent a treatment-refractory subgroup.

### Complement activation defines a protein signature with high disease severity

**Fig. 3:**
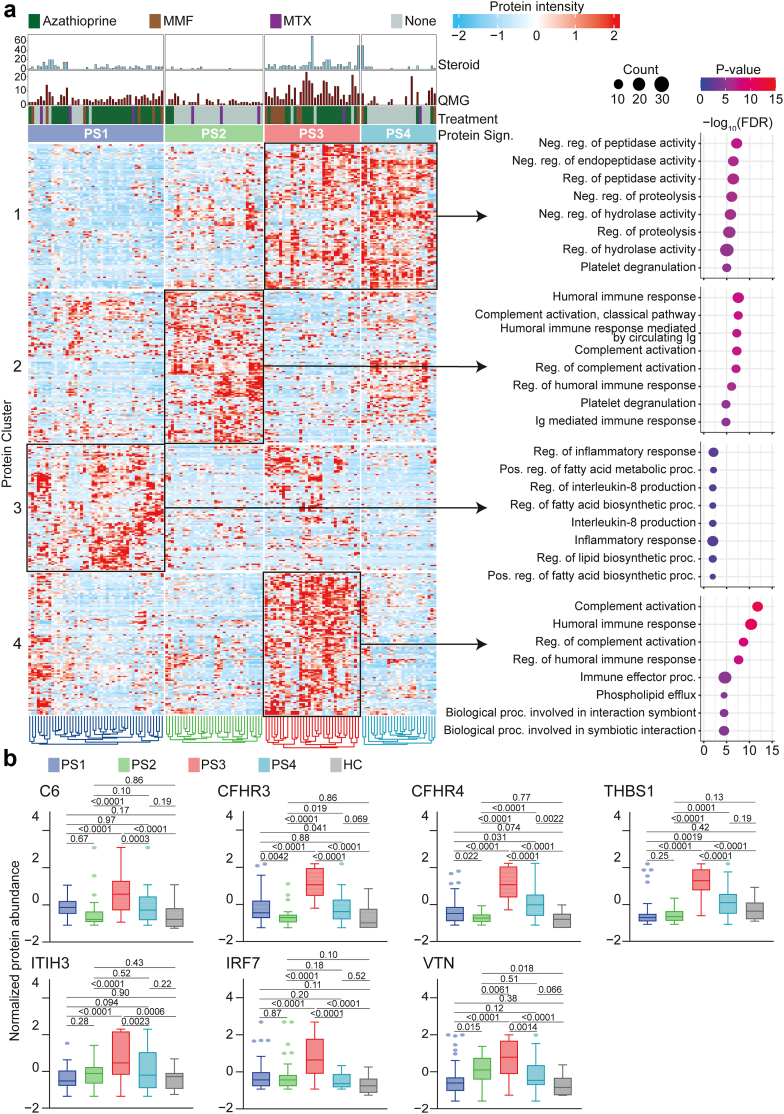
Functional enrichment analysis of protein signatures. (a) Heatmap as shown in Fig. 1. Each protein cluster is indicated by a black box. All proteins constituting a cluster were analysed for enrichment for the biological processes (BP) gene ontology with each cluster analysed separately. Negative decadic logarithms of corresponding P-values are depicted on the x-axis. Counts of associated proteins are illustrated by circle sizes. (b) Individual proteins constituting protein cluster 4 shown as box plots. Whiskers extend from the box to the minimum and maximum values within 1.5 × IQR from Q1 and Q3, respectively. Groups were compared by the ordinary one-way ANOVA test. CFHR, complement factor H related protein; IRF7, interferon regulatory factor 7; THBS1, thrombospondin-1; VTN, vitronectin.

Next, we asked whether the observed protein signatures may reflect meaningful biological pathways. To this end, we performed enrichment analysis for each protein cluster (Fig. 3a). Each patient phenotype was defined by protein clusters with different gene ontology (GO) terms. For example, protein cluster 1 was shared between the PS3 and PS4 group and was associated with negative regulation of peptidase activity. Protein cluster 2, which defined PS2, comprised elements of the humoral immune response and classical complement activation, among others. Interestingly, while the group of PS3 patients shared protein cluster 1 with group PS4, engagement of protein cluster 4 was only observed in PS3 patients and therefore specific for these patients. This PS3-specific protein cluster was defined by pronounced complement activation and the humoral immune response. Given the high disease severity and concurrent enrichment for complement activation in PS3, we focused on this patient subtype. First, we manually screened the 20 most strongly enriched proteins for the PS3 group to better understand their protein pattern. In line, complement proteins, such as complement component 6 (C6), complement factor H related 3 (CFHR3) or complement factor H related 4 (CFHR4), were more abundant in PS3 patients than in the other groups (Fig. 3b). Concurrently, these patients also demonstrated high levels of complement-associated proteins, such as thrombospondin 1 (THBS1), inter-alpha-trypsin inhibitor heavy chain H3 (ITIH3), interferon regulatory factor 7 (IRF7) and vitronectin (VTN).

Succinctly, consensus clustering identified a distinct subtype of MG characterised by markers of complement activation in the serum. While all patients shared Abs against the AChR, we hypothesise that these Abs may differ in their potency for complement induction, and, thus, inducing the observed pattern in the peripheral blood.

### Protein signature 3 is characterised by an hyperexpanded antibody repertoire

Following this line of argumentation, we sought to further characterie each PS based on their Ab architecture. For this purpose, we selected three patients from each PS for in-depth immunogenomic analysis. To exclude the impact of differences in treatments, we sampled three patients from each PS that were treatment naïve at the time of sampling. In PS2 and PS4, only three patients received azathioprine and were thus selected. In PS1 and PS3, three patients were randomly selected from all azathioprine-treated patients. Previously, PBMCs had been collected at baseline (together with the serum samples) and stored for BCR analysis.

**Fig. 4:**
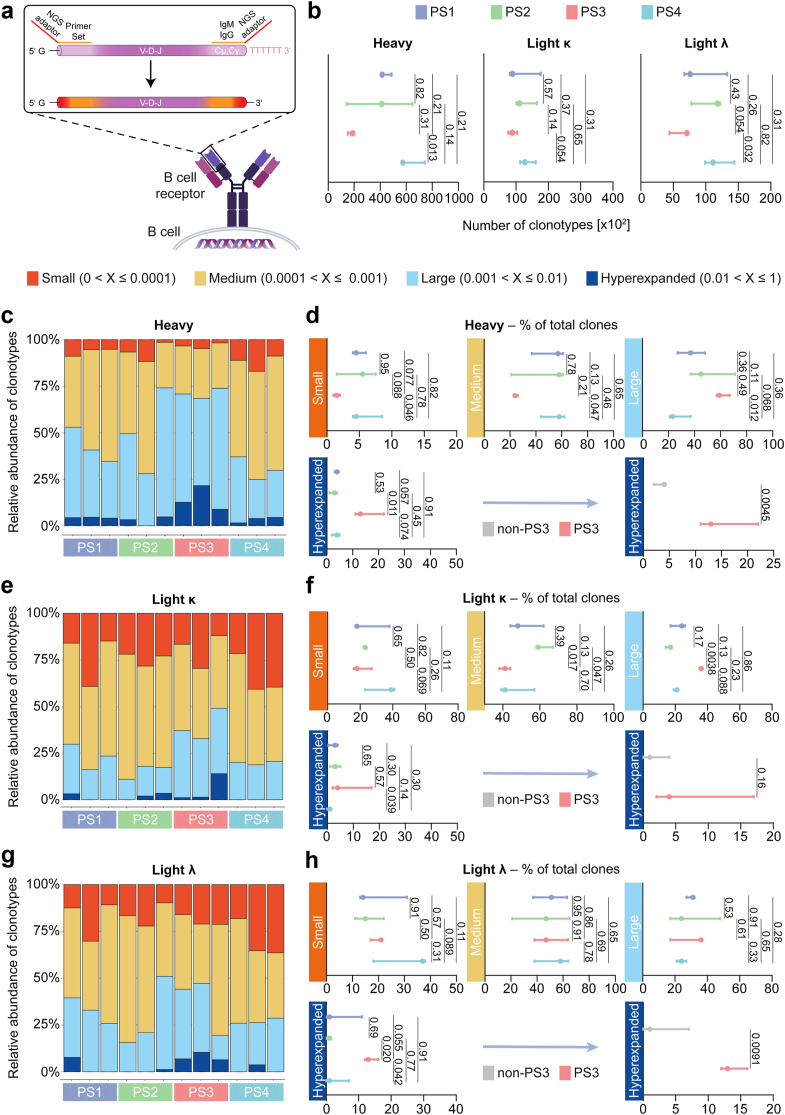
Immunogenomic analysis of the B cell receptor repertoire. (a) Overview of the immunogenomic analysis. The V-D-J segment was amplified using NGS adaptors. The BCR repertoire was analysed for 12 patients with n = 3 per group. (b) Number of clonotypes for the heavy chain and the kappa and lambda light chains of the IgG isotype. (c) Relative frequency of specific clones occupying the indicated proportion of the total BCR repertoire for the IgG heavy chain. Each stacked bar refers to one patient. (d) Comparison of relative frequencies of the indicated clonotypes between groups for the IgG heavy chain. (e) Relative frequency of specific clones for the κ light chain. (f) Comparison of relative frequencies of the clonotypes between groups. (g) Relative frequency of specific clones for the λ light chain. (h) Comparison of relative frequencies of the clonotypes between groups. Differences between groups were determined by the Kruskal–Wallis test. Error bars indicate the median with 95% confidence intervals.

Briefly, the V(D)J sequence of the BCR was amplified by short-read amplicon sequencing. Heavy and light chains (kappa (κ) and lambda (λ)) were amplified separately and sequenced (Fig. 4a). For downstream analysis, we focused on the immunoglobulin (Ig) G subtype as pathogenic anti-AChR-Abs are most likely derived from this type of Ig.^1^^,^^4^ We first computed the clonality of each patient’s repertoire stratified by the corresponding PS phenotype. Here, the number of heavy and light chain clonotypes were lower for the PS3 group compared to other groups (Fig. 4b). A lower number of clonotypes may be due to the expansion of specific BCR clonotypes occupying a large portion of the repertoire. Indeed, while hyperexpanded clones occupied between 0 and 5% of the heavy chain repertoire in PS1, PS2 and PS4, patients in PS3 demonstrated hyperexpansion in 10–20% of their clonal repertoire (Fig. 4c and d). For the κ-light chain, one PS3 patient displayed marked hyperexpansion, while all other patients had comparable frequencies of hyperexpanded κ-light chains (Fig. 4e and f). Analysis of the λ-light chain revealed that the BCR repertoire of PS3 patients harboured around 10% of hyperexpanded clonotypes, while other groups of PS had lower frequencies (Fig. 4g and h).

Further, we analysed the Ig distribution across the BCR repertoire (Supplementary Fig. S4a). Here, Ig usage was comparable across PS groups with the heavy chains IgA and IgM being most frequent. To study the V(D)J rearrangement of the BCR, we compared the gene usage across PS groups. Usage of the Ig heavy chain variable region (IGHV) demonstrated no differences between PS groups (Supplementary Fig. S4b). IGHV4.39, IGHV3.23 and IGHV4.59 were the three most frequently used IGHV genes. For the Ig heavy chain joining (IGHJ) gene, the BCR repertoire of all patients was mostly composed of the IGHJ4 gene (Supplementary Fig. S4c), as previously reported.^28^ Finally, we computed the V-J arrangement of the IGHV and IGHJ genes for each patient and group. Interestingly, only PS3 had the IGHV3.7/IGHJ4 pair as top V-J frequency, while all PS shared IGH3.23/IGHJ4 as frequent pairing (Supplementary Fig. S5). Taken together, PS3 is characterised by hyperexpanded BCR clones and a skewed usage of the IGHV genes. After recognition of its cognate antigen, B cells undergo clonal expansion, thereby increasing the Ab-binding affinity of their BCR clone to its respective antigen.^29^ High affinity binding of the AChR could provide a link between the skewed Ab repertoire of PS3 patients and their increased disease severity.

### Complement induction is amplified in protein signature (PS) 3 *in vitro*

**Fig. 5:**
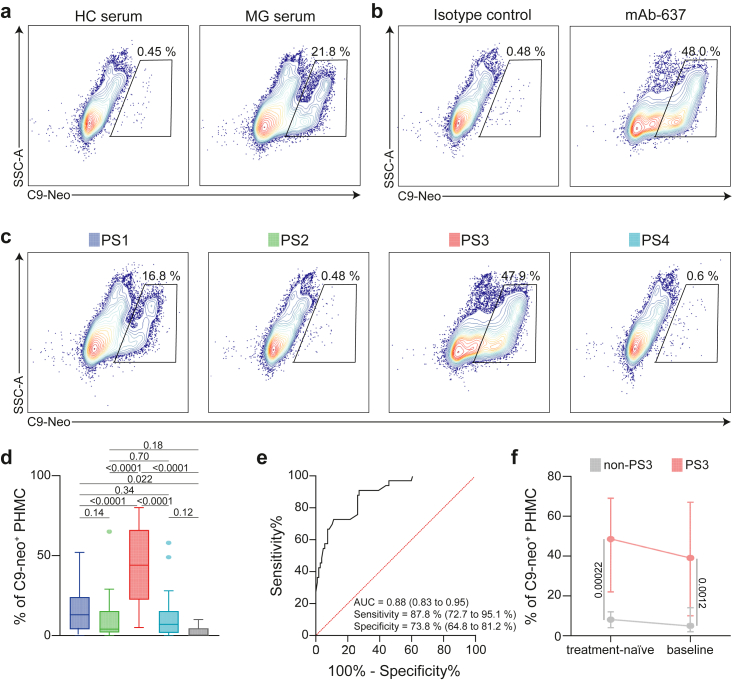
Cell-based assay for membrane attack complex formation. (a) Representative flow cytometry gating for live PHMC treated with serum from patients diagnosed with MG or HC. The percentage of PHMC positive for the C9-neoantigen is indicated. (b) Representative flow cytometry gating for live PHMC treated with an isotype control or mAb-637. (c) Representative flow cytometry gating for live PHMC treated with serum from different PS groups. (d) Flow cytometric analysis of C9-neoantigen formation on PHMC treated with serum from each PS group. Differences between groups were determined by the ordinary one-way ANOVA test. Bar plots indicating the median with 95% CI. (e) Receiver operating characteristic curve (ROC) for classification of PS3 and non-PS3 patients based on levels of MAC formation. The AUC, specificity and sensitivity are indicated with their 95% confidence intervals in brackets. (f) Flow cytometric analysis of C9-neoantigen formation on PHMC treated with serum from patients at study baseline and from a treatment naïve timepoint earlier later stratified as PS3 (n = 11) and non-PS3 (n = 17). Error bars indicate the median and the 95% confidence intervals.

To understand whether the PS3 phenotype harbours anti-AChR-Abs with higher affinity for its antigen and, consequently, the potency for complement activation, we aimed to study Ab-mediated complement MAC formation *in vitro*. Briefly, we modified a previously reported cell-based assay for the measurement of MAC formation. Here, AChR-expressing cells are incubated with patient sera and MAC formation is quantified by flow cytometry.^30^ For the current study, we aimed to provide a closer approximation to the human system. To overcome limitations of immortalised cell lines, we chose PHMCs as source of AChR. These cells differentiate *in vitro* and form neuromuscular junctions (NMJs) resembling the complex topography of skeletal muscle *in vivo*.^31^^,^^32^ To test the integrity of this platform, differentiated PHMCs were incubated with complement-competent normal human serum (NHS) and serum from MG or that of HCs (Fig. 5a). These were the same serum samples as previously used for the proteomics analysis. We used NHS to ensure that complement factors are present in each experiment. As readout, we measured the levels of a complement component 9 (C9) neo-epitope indicating MAC formation by flow cytometry. Incubation with MG serum resulted in MAC formation, but not incubation with HC serum. Further, we validated this setup by treating the cells with an AChR-specific human recombinant IgG1 subclass monoclonal Ab (mAb-637)^30^ and an isotype control (Fig. 5b). The mAb-637 resulted in MAC formation while the isotype control did not, suggesting that this platform enables measurement of anti-AChR-Ab-mediated complement activation.

Next, we tested MAC formation for all 140 MG serum samples analysed in this study in the aforementioned setup. Samples were measured concurrently to prevent a batch effect. Here, serum samples from the PS3 subtype of patients resulted in higher levels of MAC formation as compared to other patients (difference between means of the PS3 and non-PS3 groups (95% CI): 31.4 (25.1–37.6), P < 0.0001, t-test) (Fig. 5c and d). We dichotomised patients into PS3 and non-PS3 to test if MAC formation allows to classify patients into these groups (AUC (95% CI)): 0.9 (0.8–1.0) Fig. 5e). The sensitivity (95% CI) was 87.8% (72.7–95.1%) with a specificity (95% CI) of 73.8% (64.8–81.2%). In line with previous reports,^30^ the potency for MAC formation was highly heterogenous across patients. In addition, we sought to determine whether observed differences between groups were influenced by variations in treatment regimens. To this end, we screened for patients with available serum samples obtained prior to the study’s baseline. Specifically, we identified individuals who were recently diagnosed and had not yet received any immunosuppressive therapy or steroids at the time of blood collection or prior. A total of 17 serum samples met these criteria, comprising 11 patients categorised later as non-PS3 and six as PS3 based on proteomic analysis. The median duration between the initial blood sampling, when patients were treatment-naïve, and the study baseline was three months (IQR 1 to 6) for the non-PS3 group and five months (IQR 2.5 to 9) for the PS3 group, demonstrating no differences (P = 0.32, Mann–Whitney U test). At the baseline assessment, seven out of 11 patients in the non-PS3 category and five out of 6 in the PS3 category were receiving azathioprine. The seven azathioprine-treated patients in the non-PS3 group also received steroids (median dose per day 5 mg (IQR 3 to 8)), while all six patients from the PS3 group were treated with steroids (median dose per day 8 mg (IQR 5 to 11.5). Analysis of these serum samples in our cell-based assay demonstrated an elevation in MAC formation in PS3 patients compared to non-PS3 patients, even at a treatment-naïve stage, and, as such, early in their disease courses and before the initiation of therapies (Fig. 5f).

Consistent with our previous findings, patients belonging to the PS3 group appear to harbour high-affinity anti-AChR-Abs that are potent complement inductors.

### Protein signature 3 identifies responders to complement inhibition

The subgroup of PS3 patients was characterised by excessive complement activation. Consequently, we suspected that these patients may be prone to respond to complement inhibition than patients without detectable complement activity. To test this assumption, we continued to observe the clinical course of patients included at baseline for a follow-up period of 24 months. From the original cohort of 140 patients, 26 were lost to follow-up. Out of the remaining patients, 16 patients were switched to a C5 complement inhibition therapy (C5IT) based on clinical indication. These therapies included eculizumab (n = 9) and ravulizumab (n = 7). The indication for the treatment switch was made by the treating physician who was independent from the study enrolment. The time between baseline and switch to C5IT was on average 8 months (standard deviation [SD] 4 months) without differences between PS groups. Clinical characteristics of this cohort are given in Table 2. We collected clinical data for three months after the treatment switch including the QMG and MG-ADL scores. Scoring was performed by the treating physician, who was blinded to the study inclusion and the PS label of each patient. Clinical data was stored centrally and assessed after completion of the three months observation period. The concurrent IST was stable for all included patients.

**Table 2:** Overview of the follow-up cohort.

Clinical characteristics	PS1	PS2	PS3	PS4
Number of patients	6	2	7	1
Gender				
Female	3	2	3	0
Male	3	0	4	1
Age (years), median (IQR, Q1–Q3)	42 (38–53)	40 (38–40)	52 (31–70)	55
QMG score, median (IQR, Q1–Q3)				
Baseline^a^	9 (3–7)	7 (6–8)	10 (6–12)	5 (0)
MG-ADL score, median (IQR, Q1–Q3)				
Baseline^a^	10 (8–12)	8 (6–10)	14 (8–16)	6
Treatment				
Azathioprine	5	2	5	1
Methotrexate	1	0	1	0
Mycophenolate-mofetil	0	0	1	0
Number of patients treated with steroids	4	2	4	1
Steroid dose/day, median (IQR, Q1–Q3)	5 (2–7)	2	5 (3–7)	2 (0)

IQR, interquartile range; MG-ADL, myasthenia gravis activities of daily living; PS, protein signature; QMG, quantitative myasthenia gravis; Q1, first quartile; Q3, third quartile.

a Baseline is defined as the time of switch to a complement inhibitor.

**Fig. 6:**
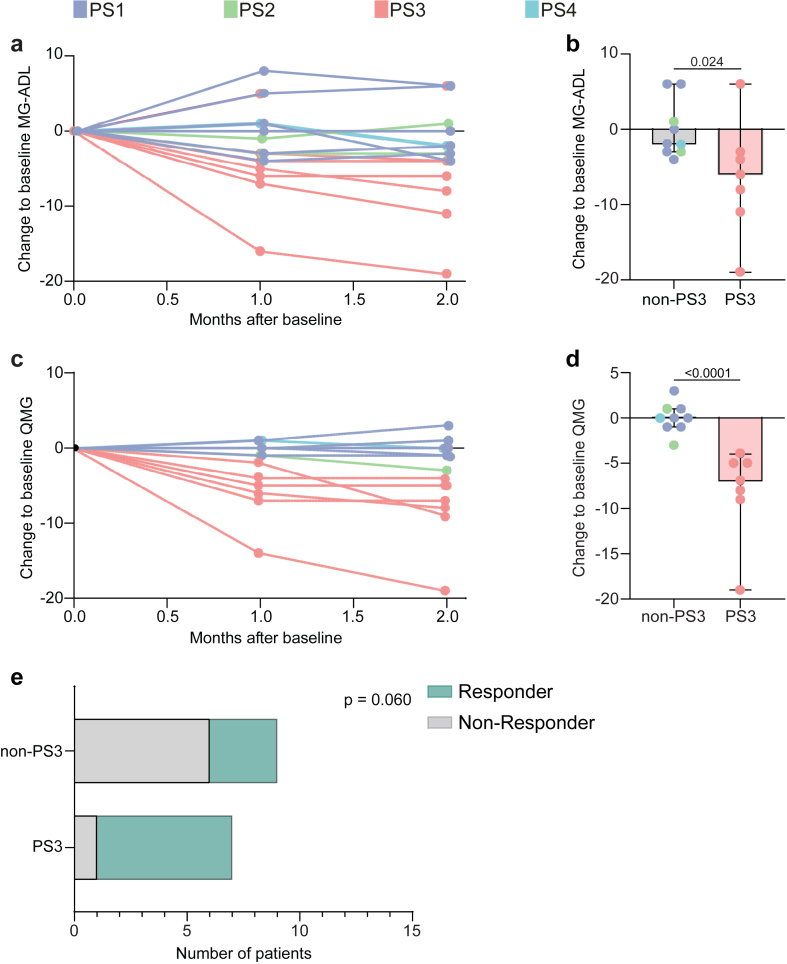
Clinical response to complement inhibition. (a) Change to baseline MG-ADL score for each individual patient. The corresponding PS is indicated by colour code. (b) Change to baseline MG-ADL score after two months of observation. Comparison between patients belonging to PS3 (n = 7) and non-PS3 (n = 9). (c) Change to baseline QMG score for each individual patient. The corresponding PS is indicated by colour code. (d) Change to baseline QMG score after two months of observation. Comparison between patients belonging to PS3 and non-PS3. Whiskers extend from the box to the minimum and maximum values within 1.5 × IQR from Q1 and Q3, respectively. Differences between groups were determined by the Mann–Whitney U test. (e) Number of patients defined as treatment responder or non-responder stratified by their PS phenotype. Differences between groups were determined by the Fisher’s exact test.

We visualised the individual trajectory of the MG-ADL score for each patient stratified by the PS group label (Fig. 6a). Comparing the response to C5IT between patients belonging to the PS3 group and those who did not, the change to the MG-ADL score after two months of treatment was higher for PS3 patients (difference between medians of the PS3 and non-PS3 groups (95% CI): 4 (0–14), P = 0.024, Mann–Whitney u test) (Fig. 6b). Similarly, QMG reduction in response to C5IT was more pronounced in the PS3 group compared to other patient subgroups (difference between medians of the PS3 and non-PS3 groups (95% CI): 7 (4–10), P < 0.0001, Mann–Whitney u test) (Fig. 6c and d). As previously employed in the REGAIN trial,^33^ a ≥3 point improvement on the MG-ADL scale was used to define treatment responder and non-responder. Patients were assigned as treatment responder and non-responder after the three-month follow-up period. Patients belonging to the PS3 phenotype were more frequently defined as treatment responder than patients not belonging to this phenotype, however, without reaching statistical significance (percentage of responder for PS3 vs non-PS3: 85.7% vs 33.3%, P = 0.061, Fisher’s exact test, Fig. 6e).

**Fig. 7:**
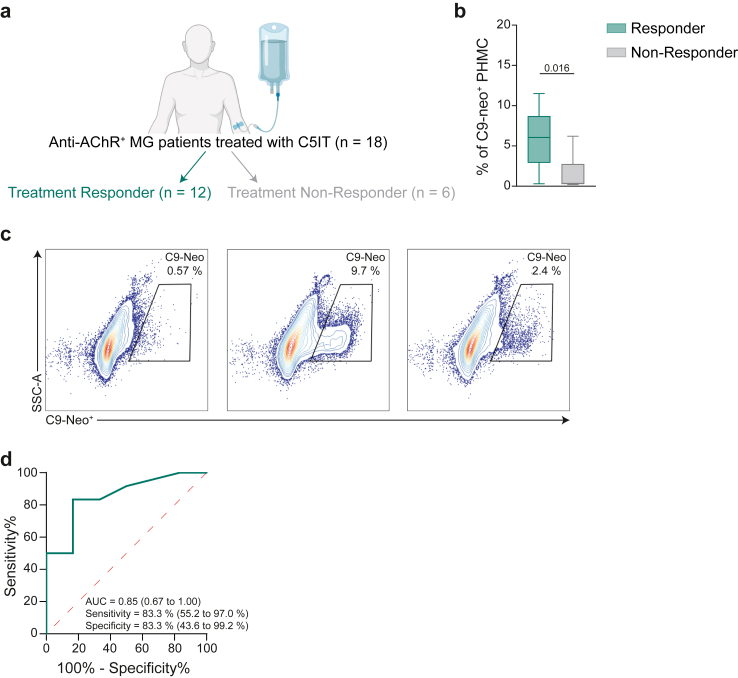
Prospective validation cohort. (a) 18 patients with anti-AChR-Ab positive MG were included and stratified into treatment responders (n = 12) and non-responders (n = 6) based on a cutoff of ≥3 point improvement on the MG-ADL scale. (b) Flow cytometric analysis of C9-neoantigen formation on PHMC treated with serum from each patient grouped into treatment responders and non-responders. Whiskers extend from the box to the minimum and maximum values within 1.5 × IQR from Q1 and Q3, respectively. (c) Representative flow cytometry gating for live PHMC treated with serum from different groups. (d) Receiver operating characteristic curve (ROC) for classification of treatment responders and non-responders based on levels of MAC formation. The AUC, specificity and sensitivity are indicated with their 95% confidence intervals in brackets. Differences between groups were determined by the two-sided Student’s T-test.

Finally, we aimed to validate these observations in an independent, prospective cohort of patients. For this purpose, we screened patients requiring a treatment switch to a C5IT in the participating centres. As before, the indication for the treatment switch was made by the treating physician independently from the study enrolment. These patients were required to not have been included in the initial cohort and to provide informed written consent before study inclusion. In total, we recruited 18 patients for this cohort (Fig. 7a). Clinical data was collected, and the individual treatment responses were recorded (Table 3). In respect to treatment at baseline, 16 patients were treated with an IST with the majority receiving azathioprine (13 patients), and two patients on methotrexate and one on mycophenolate-mofetil. The two patients without IST were on steroid treatment at baseline, as well as 9 out of 16 IST-treated patients. The individual treatment strategies and steroid doses per group at baseline are detailed in Table 3. The observation period was three months in total. At baseline (time of treatment switch), serum was collected from all patients and stored for the duration of the follow-up period. After completion, we employed our cell-based assay, as described above, to determine whether the patient’s serum induces complement activation on PHMCs. This choice was motivated by the ability of the cell-based assay to effectively identify patients belonging to the PS3 phenotype and because we believe that the assessment of complement activation in a primary muscle cell culture is a close approximation to the biological system. In this design, treatment responders demonstrated more profound MAC formation than treatment non-responders in the cell-based assay (difference between medians of the PS3 and non-PS3 groups (95% CI): 4.3 (0.9–7.7), P = 0.016, Mann–Whitney U test, Fig. 7b and c). Consistently, MAC formation was able to classify treatment responders and non-responders at baseline (AUC (95% CI): 0.9 (0.7–1.00), Fig. 7d). Here, the sensitivity (95% CI) was 83.3% (55.2–97.0%) with a specificity (95% CI) of 83.3% (43.6–99.2%).

**Table 3:** Overview of the validation cohort.

Clinical characteristics	Prevalence
Anti-AChR-Ab-positive, n (%)	18 (100%)
Gender	
Female	12
Male	6
Age (years), median (IQR Q1–Q3)	64 (38–70)
Thymoma, n	
No thymoma	17
Thymoma	1
QMG score, median (IQR, Q1–Q3)	
Baseline^a^	9 (6–12)
MG-ADL score, median (IQR, Q1–Q3)	
Baseline^a^	10 (5–13)
Treatment	
Treatment naïve	1
Standard IST	17
Azathioprine	9
Methotrexate	1
Mycophenolate-mofetil	2
Number of patients treated with steroids, n (%)	14
Steroid dose/day, median (IQR, Q1–Q3)	17 (8–25)

IQR, interquartile range; MG-ADL, myasthenia gravis activities of daily living; PS, protein signature; SD, standard deviation; QMG, quantitative myasthenia gravis; Q1, first quartile; Q3, third quartile.

a Baseline is defined as the time of switch to a complement inhibitor.

Collectively, this data suggests that patients belonging to the PS3 phenotype are more likely to benefit from complement inhibiting therapies. Moreover, evaluating MAC in a cell-based assay could act as a surrogate marker for identifying these patients consequently improving the precision of treatment allocation.

## Discussion

In this study, we provide a proteomic classification of MG that employs consensus clustering to identify meaningful patient phenotypes based on protein patterns. The advantage of consensus clustering lies in its ability to overcome the limitations of individual clustering algorithms. By combining multiple algorithms and assessing their level of agreement, consensus clustering can reduce the impact of algorithmic biases and provide more robust and reliable subgroup identification.^19^ This approach has proven successful for the identification of proteomics-based cancer patient subgroups, among others.^34^ Here, we extend this strategy to the study of MG as prototypical autoimmune disease. Analysis of the serum proteome delineates a distinct patient subgroup characterised by severe disease, a hyperexpanded BCR repertoire and preferable response to complement inhibition. In this specific study cohort, it’s noteworthy that the MG-ADL and QMG scores exhibited a strong correlation, a finding consistent with prior research indicating varying degrees of association between these scales^35^^,^^36^ with patients responsive to treatment demonstrating the highest level of correlation.^37^

We suspect that if complement-mediated damage to the NMJ is severe enough, this damage is reflected in the peripheral blood. Given the clinical implications, a number of studies have addressed this link in the past.^30^^,^^38^, ^39^, ^40^ However, most studies did not observe a correlation between complement factors and disease activity.^30^^,^^38^, ^39^, ^40^ Complement is a complex serum-effective system with high inter-individual variability.^41^ While the interaction between Abs and their cognate antigens constitutes the focus point of Ab-mediated complement activation, various context factors including epitope specificity, Ab-affinity, IgG subclass usage and post-translational modifications alter the efficacy and trajectory of downstream complement induction.^30^^,^^41^^,^^42^ Following this line of argumentation, individual anti-AChR-Abs that recognise a single subunit of the AChR are often unable to effectively induce complement-mediated tissue damage.^43^ In contrast, the synergy between Abs recognising different AChR epitopes triggers profound complement activation *in vitro* and *in vivo*.^43^ This may provide an explanation why measuring a single complement factor or cleavage product is unlikely to capture the degree of complement activation at the target structure. The current study may overcome this caveat as the key advantage of proteomics-based clustering is its ability to recognise patterns. These patterns allow to assign patients to a biological signature defined by a protein network reflecting complement activity, instead of relying on single protein markers. The proposed classification also confers pathophysiological insights. The recognition of their cognate antigens results in the clonal expansion of the corresponding B cells.^44^ During this expansion, the insertion of random mutations drives the accumulation of high-affinity B cell clones and the diversification of the Ab repertoire. In this study, we observed that a subtype of patients harboured a clonally hyperexpanded BCR repertoire capable of effectively inducing MAC formation. We suspect that the degree of clonal hyperexpansion is linked to the formation of high-affinity B cell clones capable of effectively engaging the AChR. This may provide an explanation why Ab titers do not correlate with disease severity^39^^,^^45^ as the current data suggests that the degree of clonal hyperexpansion and the affinity between the anti-AChR-Ab and its antigen may define complement-mediated tissue damage. Indeed, besides assessing complement activation markers, studies have increasingly turned to cell-based assays to understand the functional consequences of Ab binding in MG. These assays leverage cell lines, such as HEK293T engineered to express AChR,^30^ to measure MAC formation *in vitro*. Consistent with findings from these investigations,^30^ we have also observed a substantial variability among individual patients regarding MAC formation. These cell-based assays might provide readouts that more closely mimic the *in vivo* situation of individual patients, potentially identifying those with highly pathogenic Abs that trigger MAC formation. Such patients could potentially benefit from complement inhibition, thus conferring clinical utility to these assays. However, the choice of the cellular target for these assays is important. While our study opted for primary cells, offering the advantage of NMJs resembling the complex topography of human muscle, there are drawbacks. Primary cells entail greater sample-to-sample heterogeneity and raise concerns regarding result reproducibility and handling. Despite the technical and logistical challenges, cell-based assays hold promise for improving the stratification of patients with MG by providing a more comprehensive assessment of complement activation and functional Ab activity. However, comparative studies are required to identify the optimal cellular target for assays measuring complement activation.

The clinical relevance of this strategy is supported by the prediction of treatment responses to complement inhibition. While further standardisation is needed, the implementation of cell-based complement assessments might provide clinical value for the management of MG by identifying patients likely to benefit from complement inhibition. Going forward, combining functional Ab-assays with a standardised set of complement-related readouts could further improve patient stratification and, thereby, patient management. Concurrently, there is a need to explore whether varying methodological approaches yield comparable results in patient stratification based on complement profiles, or if they offer distinct outcomes and biological insights. Alternative high-throughput strategies, such as (bulk)-transcriptomics, could be leveraged to address this question.

The study is subject to limitations, notably the relatively small sample size of patients undergoing C5IT during the follow-up period, as well as the restricted number of patients accessible for immunogenomic analysis. Additionally, differences in treatment strategies among patient groups, inherent to the cross-sectional design, pose a potential confounder. Patients with more severe disease may receive higher doses of steroids, ISTs, or a combination thereof. These variations could influence proteomic profiles within specific patient groups or subgroups. Despite our efforts to align patients based on treatment strategies, it’s important to consider this potential confounder when interpreting the data. Prospective studies focusing on a treatment-naïve or recently diagnosed group of patients are required to exclude this confounder. Finally, our study is limited to the anti-AChR-Ab serological group. We acknowledge that distinct proteomic patterns are expected in other serological groups, including seronegative patients or those with anti-MuSK-Abs. Further investigations are needed to study how the proteome of these patients differs and to derive meaningful biological insights. However, due to the rarity of these subgroups, substantial collaborative efforts across multiple centres will be essential to conduct such studies effectively.

Nonetheless, our study supports a link between disease severity and proteomic patterns of complement activation in MG. This knowledge may improve our molecular understanding of the disease and inform patient classification based on biological signatures.

## Contributors

Conceptualization: CN, CBS, AM, SGM, TR.

Data curation: CN, CBS, FS, AM, SGM, TR.

Formal analysis: CN, CBS, SB.

Funding acquisition: CN, CBS, SGM, TR.

Investigation: CN, CBS, SB, SW.

Methodology: CN, CBS, SB.

Project administration: CN, CBS.

Resources: AM, SGM, TR.

Software: CN, CBS, SB.

Supervision: SGM, TR.

Validation: CN, CBS.

Visualization: CN, CBS, NH, LM.

Writing—original draft: CN, CBS.

Writing—review & editing: SB, FS, LM, LT, NH, SW, DC, VD, AV, MP, SR, FFK, TS, HPH, SK, AR, AM, SGM, TR.

All authors read and approved the final version of the manuscript. CN, CBS, SB, SGM and TR accessed and verified the underlying data.

## Data sharing statement

Spectral raw proteome data were deposited in the PRIDE/ProteomeXchange repository (http://www.ebi.ac.uk/pride) and are available with identifier PXD040786 (username: reviewer_pxd040786@ebi.ac.uk; password: oL69QrhV). The full heatmap and underlying data are publicly available as interactive web server at https://masanneck.shinyapps.io/Cola-Heatmap/. The immunogenomic raw data is publically available with the following identifier: SRA accession code PRJNA1012710.

## Declaration of interests

CN, CBS, SGM and TR have been granted a patent by the European Patent Office (EPO) relating to the use of ITIH3 as a biomarker for (assessing) disease activity in myasthenia gravis patients (EP22195296.3).

CN received honoraria for lectures from Alexion, ArgenX and UCB Pharma. CBS received honoraria for lectures from Merc and travel expenses from Alexion. FS received travel support, honoraria for lectures and adboards from Alexion, ArgenX and UCB Pharma. LM received honoraria for lectures from ArgenX and travel support from Alexion. NH received honoraria for lectures and travel support from Alexion, ArgenX and Merck. MP received consulting fees, honoraria for lectures and adboards from Alexion and ArgenX. SR received honoraria for adboards and travel support from Alexion, Bristol Myers Squibb and Merck. FK received travel support from Alexion, Merck and Novartis. TS received grants from Alnylam Pharmaceuticals, CSL Behring, Novartis, Siemens; TS received consulting fees, honoraria for lectures and adboards from Alexion, Alnylam Pharmaceuticals, argenx, Bayer Vital, Biogen, Bristol Myers Squibb, Celgene, Centogene, CSL Behring, Euroimmun, Grifols, Hexal AG, Horizon, Janssen-Cilag, Merck Serono, Novartis, Pfizer, Roche, Sanofi, Siemens, Swedish Orphan Biovitrum, Teva, Viatris. AM received consulting fees from Alexion (AstraZeneca Rare Disease), Argenx, Janssen Pharmaceuticals, Merck, Octapharma, Union Chimique Belge (UCB), Xcenda; AM received honoraria for lectures and adboards from Alexion, Argenx, Desitin, Dierks, Grifols, Hormosan, Novartis, Sanofi. SGM received honoraria for lectures and travel support from Academy 2, Argenx, Alexion, Almirall, Amicus Therapeutics Germany, Bayer Health Care, Biogen, BioNtech, BMS, Celgene, Datamed, Demecan, Desitin, Diamed, Diaplan, DIU Dresden, DPmed, Gen Medicine and Healthcare products, Genzyme, Hexal AG, IGES, Impulze GmbH, Janssen Cilag, KW Medipoint, MedDay Pharmaceuticals, Merck Serono, MICE, Mylan, Neuraxpharm, Neuropoint, Novartis, Novo Nordisk, ONO Pharma, Oxford PharmaGenesis, QuintilesIMS, Roche, Sanofi-Aventis, Springer Medizin Verlag, STADA, Chugai Pharma, Teva, UCB, Viatris, Wings for Life international and Xcenda. TR received honoraria for lectures, adboards and travel support from Alexion, Argenx, Biogen, Celgene, BMS, Genzyme, Merck Serono, Novartis, Novartis, Roche, Sanofi-Aventis and Teva.

All other authors declare that they have no competing interests.
