# Supplementary material for: Identification of disease phenotypes in acetylcholine receptor-antibody myasthenia gravis using proteomics-based consensus clustering
Source: eBioMedicine. 2024 Jul 2;105:105231. doi: 10.1016/j.ebiom.2024.105231 (PMC11269806; doi:10.1016/j.ebiom.2024.105231)
Supplement: Supplementary Figs. S1–S5 [file mmc1.docx]

**Supplementary Figures**

**
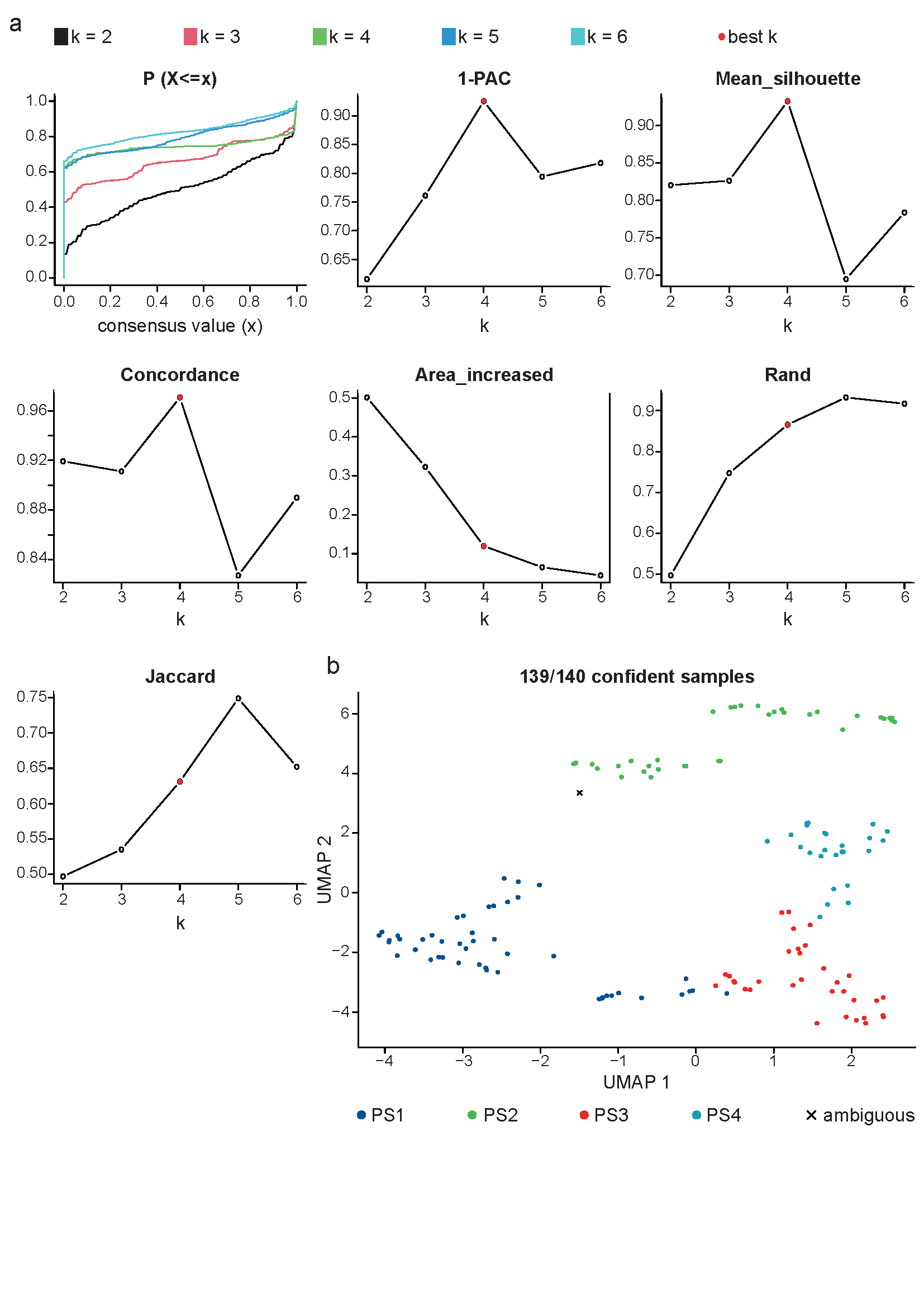
**

**Fig. S1**. **Consensus clustering of the proteomic dataset. (a)** Readouts to determine the optimal consensus clustering. Colours indicate the different levels of partitioning (here denotated as k). A number of partitioning between one and six was tested. The optimal partitioning (k) is indicated by a red dot. **(b)** Uniform manifold approximation and projection (UMAP) for k = 4 consensus clustering. 139 of 140 samples were assigned confidently.

*PAC, probably approximately correct; PS, protein signature; UMAP, Uniform manifold approximation and projection.*

*
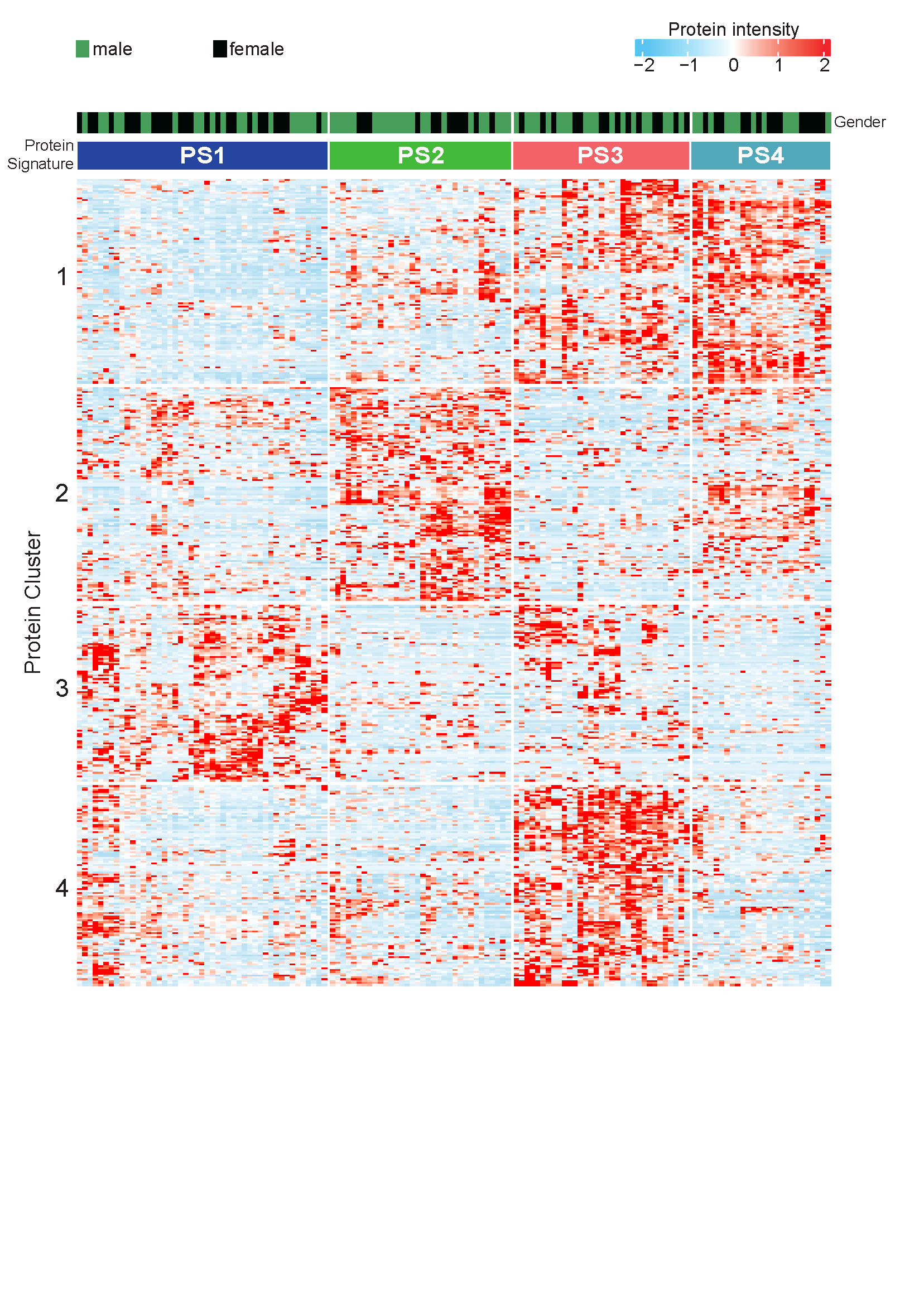
*

**Fig. S2**. Consensus-clustering disaggregated by gender. The gender is superimposed on the heatmap.


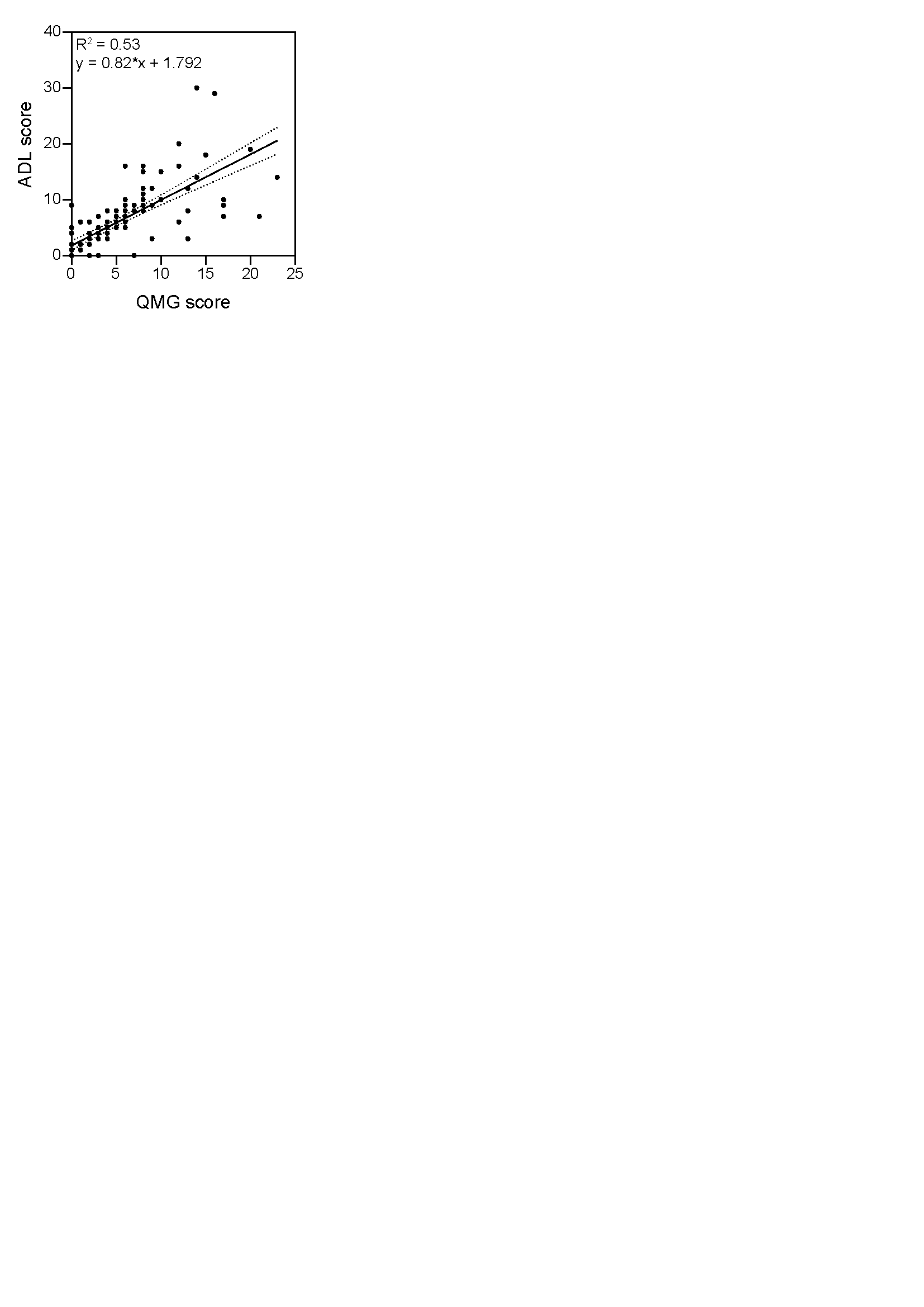


**Fig. S3**. **Linear regression of the QMG and MG-ADL scores.** The dotted line indicates the 95% confidence interval. R^2^ indicates the coefficient of determination.


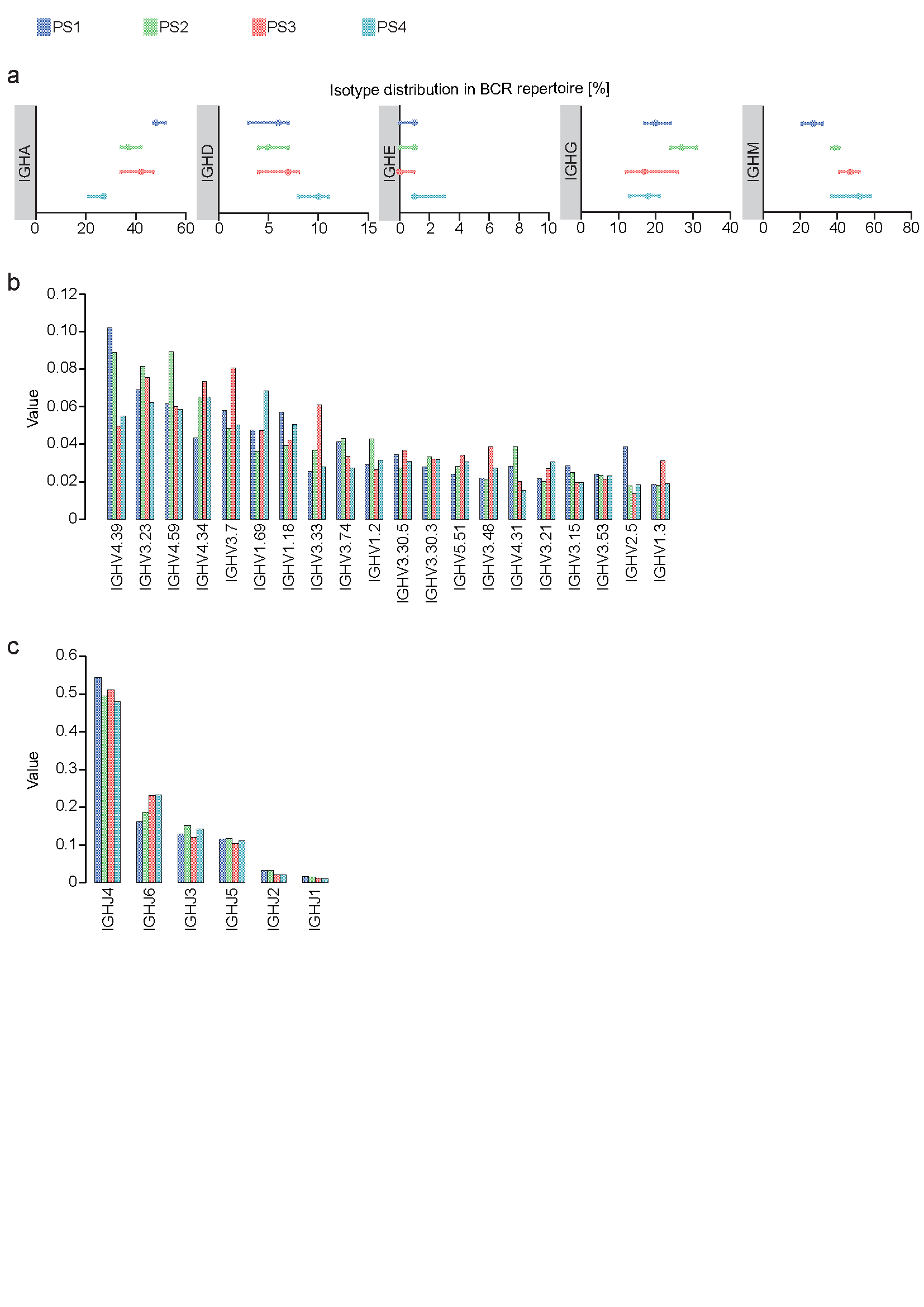


**Fig. S4**. **Isotype distribution and frequency of IgG V and J gene usage across the B cell receptor repertoire. (a)** Isotype distribution in BCR repertoire among PS1, PS2, PS3, and PS4 patient groups. Grouped bar plots depicting the relative abundance of different immunoglobulin isotypes (IgG, IgM, IgA, IgD, and IgE) in the BCR repertoire of patients. **(b)** Grouped bar plots displaying the frequency of the top 20 IgG variable (V) genes among four patient groups in the B-cell receptor repertoire. **(c)** IgG J gene usage in the BCR repertoire of PS1, PS2, PS3, and PS4 patient groups. Grouped bar plots indicating the frequency of IgG joining (J) genes among four patient groups in the B cell receptor repertoire. Error bars indicate the 95% confidence interval.

*BCR, B cell receptor; IgG, Immunoglobulin G; IgM, Immunoglobulin M; IgA, Immunoglobulin A; IgD, Immunoglobulin D; IgE, Immunoglobulin E.*

**
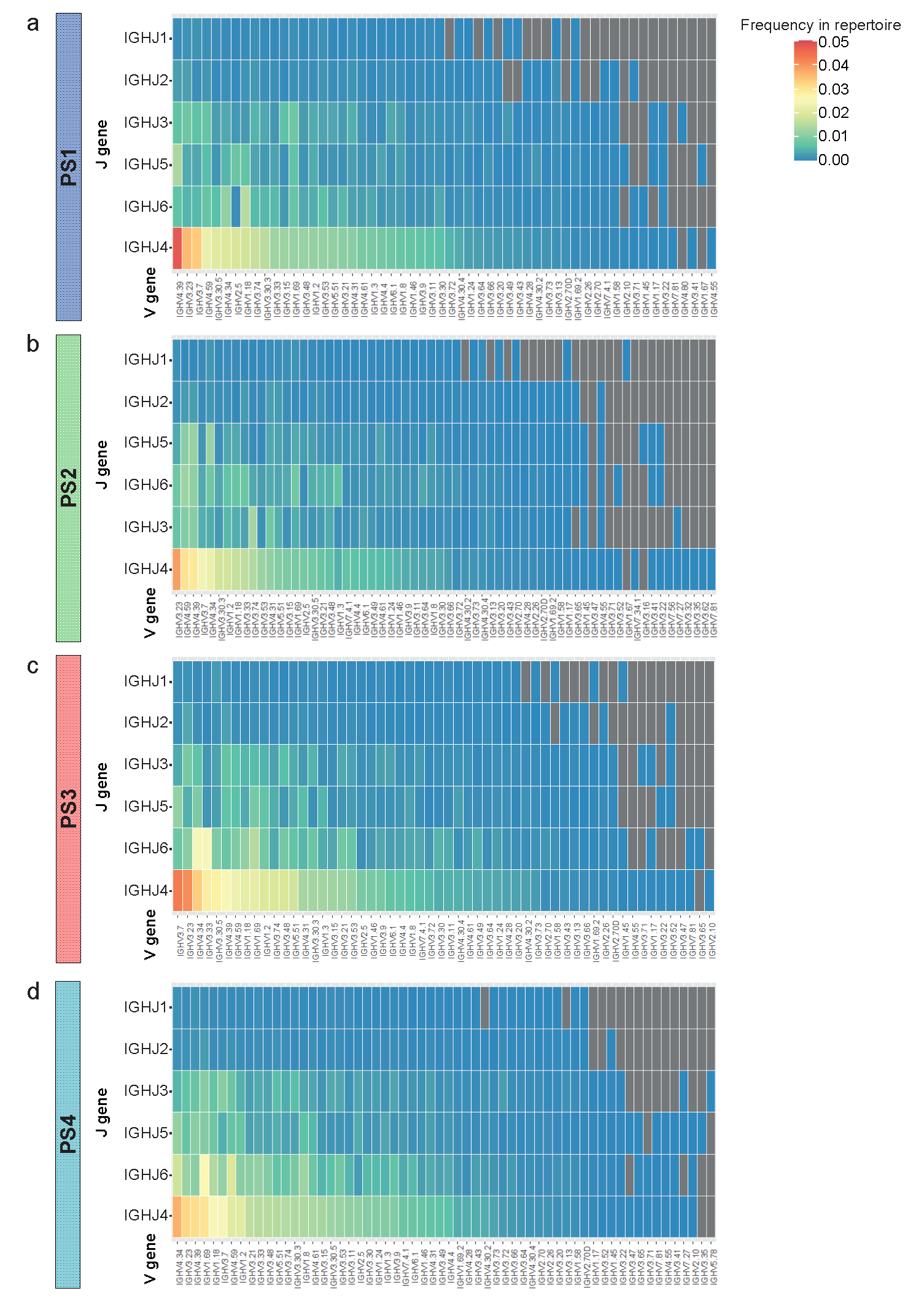
**

**Fig. S5**. **VJ gene usage distribution in PS1, PS2, PS3, and PS4 patient groups.** Heatmap representation of the IgG variable (V) and joining (J) gene combinations of the BCR repertoire in **(a)** PS1 patients, **(b)** PS2 patients, **(c)** PS3 patients, and **(d)** PS4 patients. The color intensity indicates the relative frequency of each VJ combination.

*BCR, B cell receptor; IgG, Immunoglobulin G; PS, protein signature.*
